# RAC3 influences the chemoresistance of colon cancer cells through autophagy and apoptosis inhibition

**DOI:** 10.1186/s12935-017-0483-x

**Published:** 2017-11-28

**Authors:** María Fernanda Rubio, María Cecilia Lira, Francisco Damián Rosa, Adrían Dario Sambresqui, María Cecilia Salazar Güemes, Mónica Alejandra Costas

**Affiliations:** 1 Universidad de Buenos Aires, Facultad de Medicina, Instituto de Investigaciones Médicas A Lanari, Buenos Aires, Argentina; 2 Instituto de Investigaciones Medicas (IDIM) Laboratory of Molecular Biology and Apoptosis, Consejo Nacional de Investigaciones Científicas y Técnicas, Universidad de Buenos Aires, Buenos Aires, Argentina; 3 Department of Gastroenterology, Instituto de Investigaciones Médicas Dr. A. Lanari, UBA, Buenos Aires, Argentina; 4 Department of Oncology, Instituto de Investigaciones Médicas Dr. A. Lanari, UBA, Buenos Aires, Argentina

**Keywords:** Colorectal cancer, RAC3, Chemoresistance, Apoptosis, Autophagy

## Abstract

**Background:**

RAC3 coactivator overexpression has been implicated in tumorigenesis, contributing to inhibition of apoptosis and autophagy. Both mechanisms are involved in resistance to treatment with chemotherapeutic agents. The aim of this study was to investigate its role in chemoresistance of colorectal cancer.

**Methods:**

The sensitivity to 5-fluorouracil and oxaliplatin in colon cancer cells HT-29, HCT 116 and Lovo cell lines, expressing high or low natural levels of RAC3, was investigated using viability assays.

**Results:**

In HCT 116 cells, we found that although 5-fluorouracil was a poor inducer of apoptosis, autophagy was strongly induced, while oxaliplatin has shown a similar ability to induce both of them. However, in HCT 116 cells expressing a short hairpin RNA for RAC3, we found an increased sensitivity to both drugs if it is compared with control cells. 5-Fluorouracil and oxaliplatin treatment lead to an enhanced caspase 3-dependent apoptosis and produce an increase of autophagy. In addition, both process have shown to be trigged faster than in control cells, starting earlier after stimulation.

**Conclusions:**

Our results suggest that RAC3 expression levels influence the sensitivity to chemotherapeutic drugs. Therefore, the knowledge of RAC3 expression levels in tumoral samples could be an important contribution to design new improved therapeutic strategies in the future.

## Background

Colorectal cancer (CRC) is one of the most commonly diagnosed cancers, and 1.23 million patients are diagnosed with CRC worldwide each year [1]. Despite recent advances in chemotherapies that have improved survival rates, patients with late-stage disease and elderly [2] still have a poor prognosis, and the overall mortality rate of CRC is approximately 40% [3–5].

Although CRC has been widely studied and the role of cancer stem cells in the persistence and expansion of this disease shows to be central, the genetic changes and molecular mechanisms underlying the development and progression of this cancer are not completely understood [6–11].

In CRC, chromosomal aberrations have been extensively analyzed by comparative genomic hybridization, and several frequently amplified regions, including 20q, have been identified [12]. Amplification of 20q has been also detected frequently in many other cancers, like breast, ovarian, gastric, bladder, and hepatocellular cancers [13, 14]. Amplification and overexpression of an oncogene have been shown to play an important role in the pathogenesis of various cancers, probably because overexpression of the amplified oncogene confers a growth advantage. Several candidate oncogenes have been isolated from 20q, including AIB1 at 20q12 [15].

Amplified in breast cancer 1 (AIB1), also known as Receptor associated-coactivator 3 (RAC3, SRC-3, ACTR, TRAM2, pCIP and NCoA3), is a member of the SRC/p160 coactivator family that also includes SRC-1 (NCoA1) and SRC-2 (GRIP1, TIF2 and NCoA2) [16]. RAC3 is highly expressed in several human cancers such as breast cancer [15], prostate cancer [17] and liver cancer [18, 19] and has been demonstrated to be a key regulator in tumor initiation, progression, metastasis and survival [18, 20, 21]. RAC3 can interact with nuclear receptors and other transcription factors to regulate the expression of their target genes involved in many signaling pathways, including ERα, E2F1, NF-κB and HER2/neu [21–24]. It has been reported that RAC3 is overexpressed in 35% of human CRC samples [25]; however, the role of RAC3 in CRC progression is still unknown.

We have previously found that RAC3 overexpression has an anti-apoptotic and anti-autophagic role [26–28], not only through its nuclear action, but also by positively regulating the activity of p38 and Akt kinases, inhibiting caspase-8 and -9 and blocking apoptosis-inducing factor-1 (AIF-1) translocation from mitochondria to the nucleus [27].

In this study we investigated the effect of RAC3 expression levels in the sensitivity to several chemotherapeutic drugs. We found that expression of this oncogene is significantly higher in some CRC cell lines. Interestingly, the sensitivity to chemotherapy treatments could be effectively improved by decreasing RAC3 expression and increasing the apoptotic and autophagic responses.

## Methods

### Patients and tissue specimens

CRC specimens tissues (n = 14) and normal tissue (n = 3) (Table 1) were obtained from Instituto de Investigaciones Médicas Dr. A. Lanari FMED-UBA, Buenos Aires, Argentina. All samples were confirmed by pathological examination, and staging was performed according to the 1997 CRC staging system of the UICC. Among the 14 CRC samples, 5 were from male patients and 9 were from female patients, with ages ranging from 68 to 91 years (median, 81.1 years). Informed consent was obtained from all patients, and this study was approved by the ethics committee of Instituto de Investigaciones Médicas Dr. A. Lanari.

**Table 1:** The correlation of RAC3 expression levels and clinical-pathological features in CRC

Variable	Number of patients	RAC3 expression (qPCR)			Fisher’s (*P*)
		< 1	10–100	> 100	
Normal tissue	3^a^	3 (100)			
Gender					0.5055
Male	5	2 (40.0)	2 (40.0)	1 (20.0)	
Female	9	1 (11.0)	6 (66.7)	2 (22.2)	
Differentiation					0.5941
Well	1	0 (0)	1 (100)	0 (0)	
Moderate	11	3 (27.3)	7 (63.6)	1 (9.1)	
Poor	2	0 (0)	0 (0)	2 (100)	
Tumor status					0.4660
T1	2	1 (50.0)	1 (50.0)	0 (0)	
T2	0	0 (0)	0 (0)	0 (0)	
T3	10	2 (20.0)	6 (60.0)	2 (20.0)	
T4	2	0 (0)	1 (50.0)	1 (50.0)	
Nodal status					0.1280
N0	9	3 (33.3)	4 (44.4)	2 (22.2)	
N1–2	5	0 (0)	4 (80.0)	1 (20.0)	
Metastasis status					1.000
M0	12	3 (25.0)	6 (50.0)	3 (25.0)	
M1	2	0 (0)	2 (100)	0 (0)	
Clinical stage					0.1923
I + II	7	3 (42.9)	4 (57.1)	0 (0)	
III + IV	7	0 (0)	4 (57.1)	3 (42.9)	

^a^correspond to 3 female patients

### Immunohistochemical staining

Paraffin-embedded tissue blocks were sectioned, deparaffinized in xylene and rehydrated for immunohistochemical staining [28]. Antigen retrieval was performed using sodium citrate. The sections were then incubated in H_2_O_2_ (3%) for 10 min, blocked in horse serum for 60 min and incubated with an anti-RAC3 antibody (Santa Cruz Biotechology, 1:100) at 4 °C overnight. After incubation with a universal biotinylated secondary antibody for 60 min, the specimens were incubated with H_2_O_2_-diaminobenzidine (DAB) until the desired staining intensity was observed. The sections were counterstained with hematoxylin, dehydrated and mounted.

### Cell culture and reagents

Human CRC cell lines HT-29 (ATCC^®^ HTB-38™), HCT 116 (ATCC^®^ CCL-247™) and LoVo (ATCC^®^ CCL-229™) were purchased from American Type Culture Collection (ATCC^®^ Manassas, VA, USA). All cells were maintained in DMEM/F12 (Invitrogen Corp., USA) supplemented with 10% fetal bovine serum (FBS) (Invitrogen Corp., USA) and cells were incubated at 37 °C in a humidified incubator containing 5% CO_2_.

Unless stated, reagents were obtained from Sigma Chemical Co. (St Louis, MO), Thermo Fisher Scientific (Waltham, MA) or Santa Cruz Biotechnology, USA.

### Plasmid construction and transfection

The short hairpin RNA (shRNA) target sequence for RAC3 (shRAC3) and scramble (control) were previously developed in our laboratory and cloned into the HuSH plasmid (OriGeneTechnologies, USA), shRAC3 sequence: GCTGCTAAGTCATCACTTCCGACAACAGA or shRAC3 (2) sequence: CCACATTGCCTCTTCGGTCTAATAGCATA (data not shown) [28]. LoVo cells were transfected with a plasmid expressing full-length cDNA of RAC3 or with empty vector (control). HCT 116 and LoVo cells were transfected using Lipofectamine 2000 (Invitrogen Corp., USA) according to the manufacturer’s protocol. Three days after transfection, the cells were incubated in selection medium containing 0.5 μg/ml Puromycin (HCT 116) or 0.5 mg/ml Geneticin (LoVo) (Invitrogen Corp., USA). After 14 days of selection, protein and mRNA expression were analyzed by immunoblotting and quantitative Real Time PCR (qPCR).

In reporter assays, HCT 116 control and shRAC3 cells were transfected with the reporter plasmid containing the NF-κB consensus binding sequence (κB-Luc) plus RSV-β-Gal as it was previously described [29].

### Primers and qPCR

Total RNA was extracted using TRIzol reagent (Invitrogen Corp., USA) and was employed to generate cDNA using Superscript III RT (Invitrogen Corp., USA) and an oligo(dT) primer. qPCR was performed using LightCycler^®^ 480 SYBR Green I Master (Roche, USA) according to the manufacturer’s protocol. For gene expression analysis, qPCR was performed by using sequence-specific primers for: RAC3 forward 5′-AAGTGAAGAGGGATCTGGA-3′ and reverse 5′-CAGATGACTACCATTTGAGG-3′, CD39 forward 5′-AGCAGCTGAAATATGCTGGC-3′ and reverse 5′-GAGACAGTATCTGCCGAAGTCC-3′.

For all analysis, GAPDH forward 5′-TCTCCTCTGACTTCAACAGC-3′ and reverse 5′-GTTGTCATACCAGGAAATGA-3′ was used as an internal control.

### Viability assays

Colorectal cancer cells lines were plated in 96-well flat bottom plates at a density of 8000 cells/well in 100 μl of medium. After 24 h, cells were stimulated with 5-fluorouracil (FUra 0–150 μM) or oxaliplatin (Oxa 0–50 μM). Cells were fixed at specific time points and the cell viability was determined by staining with 0.5% crystal violet. Absorbance of surviving stained cells was measured at 570 nm. The percentage of surviving cells was determined with respect to basal conditions (without any treatment).

Half maximal inhibitory concentration 50 (IC_50_) values were calculated with GraphPad Prism software (GraphPad Software Inc., USA) using the sigmoidal dose–response function. Assays were carried out in triplicate and at least three independent experiments were conducted.

### Western blot analysis

HT-29, LoVo wt, control or RAC3, HCT 116 wt, control and shRAC3 cells were harvested and lysed in RIPA buffer with protease inhibitors [29]. Then, cell lysates were separated via 6% SDS-PAGE and transferred to nitrocellulose membrane. The membranes were blocked in 10% skim milk and incubated with anti-RAC3 (Santa Cruz Biotechnology, USA).

For apoptosis experiments, cells were stimulated with FUra (3.5 μM) or Oxa (0.4 μM) for 6 or 24 h. For Western blot of LC3II/I, cells were pre-incubated with 10 μg/ml E64D and pepstatin A lysosomal protease inhibitors, before incubation with FUra (3.5 μM) or Oxa (0.4 μM) for 90 min. Then, cells were lysed as described before. Samples were separated by 10 or 15% SDS-PAGE and electro-transferred to nitrocellulose membranes. Membranes were blocked in 10% skim milk and incubated with anti-pro-Caspase 3, Beclin 1 and LC3 antibodies.

The anti-Tubulin antibody was used as an internal control (Santa Cruz Biotechnology, USA) in all the assays.

Subsequently, all membranes were incubated for 1 h with horseradish peroxidase-conjugated secondary antibody, and the specific bands were visualized by autoradiography using the chemiluminescence luminol reagent (Santa Cruz Biotechnology, USA).

### Microscopy and immunofluorescence assays

HT-29, LoVo wt, control or RAC3, HCT 116 wt, control and shRAC3 cells were seeded in 24-well plates on 12 mm glass coverslips. After 24 h, cells were stimulated with FUra (3.5 μM) or Oxa (0.4 μM) for 1, 6 or 24 h. For acetylated proteins assays, cells were pre-treated with a deacetylase inhibitor, Trichostatin A (0.4 μM TSA) and after 30 min stimulated with 0.4 μM Oxa or 3.5 μM FUra for 6 or 24 h.

For immunofluorescence assays the cells were fixed with 3% formaldehyde and 0.02% glutaraldehyde for 15 min. Incubation with primary antibody against Lys-acetylated (Cell Signaling Technology, Danvers, MA, USA) were performed 1 h room temperature in PBS with 10% FBS. Then, cells were washed with PBS, incubated with a TRITC-labeled secondary antibody for 1 h, washed with PBS, mounted on glass slides with PBS/Glycerol 1:1 solution.

Some cultures were stained with ethidium bromide (EtBr) and the morphology of death and surviving cells was observed by fluorescent microscopy. Ethidium bromide only enters into non-viable cells and stains chromatin with dark orange color [30].

Autophagy induction was monitored by monodancyl cadaverine (MDC) staining and the percentage of cells showing an aggregated stain was determined by counting a minimum of 100 cells per slide using fluorescence microscopy.

For all the assays cells were analyzed with an Olympus BX51 fluorescent microscope and 100 cells per field were counted. Images were taken with a digital camera and analyzed with NIH-ImageJ software.

### Analysis of Gene Expression Omnibus (GEO)

To compare the RAC3 expression levels between metastatic and primary lesions, in patients sensitive or not to Folic acid-5-fluorouracil and oxaliplatin treatment (FOLFOX), we used values obtained from GSE28702 data bank, platform GPL570 Affymetrix (Santa Clara, CA, USA) [31].

### Luciferase assays

HCT 116 cells were plated in 24-well plates 24 h prior to transfection at a density of 250,000 cells/well. Cells were transiently transfected with a total of 0.5 μg of DNA (including 75 ng of κB-luc and 50 ng RSV-β Gal vectors) using Lipofectamine 2000 protocol as previously reported [29]. The medium was replaced after 5 and 24 h later cells were pre-incubated with NF-κB inhibitor Sulfasalazine (SSZ 0.5 mM) for 30 min before stimulation with 3.5 μM FUra or 0.4 μM Oxa.

The assays for luciferase and β-galactosidase activity were performed after 24 h of treatment using the appropriate substrates in accordance with the manufacturer’s protocols (Promega corp.). To achieve transfections with a constant amount of DNA, appropriate amounts of empty vector (pRC3.1) were added to each well.

### Statistical analysis

At least three independent experiments were carried out in all cases. Results were expressed as the mean ± SD. The significance of differences between experimental conditions was determined using ANOVA and the Tukey Multiple Comparisons Test for paired observations and a p < 0.01 was considered statistically significant. For patients, differences in a given variable between groups were assessed using Fisher’s exact test and a p < 0.05 was considered statistically significant.

## Results

### RAC3 expression levels in CRC patients

**Fig. 1:**
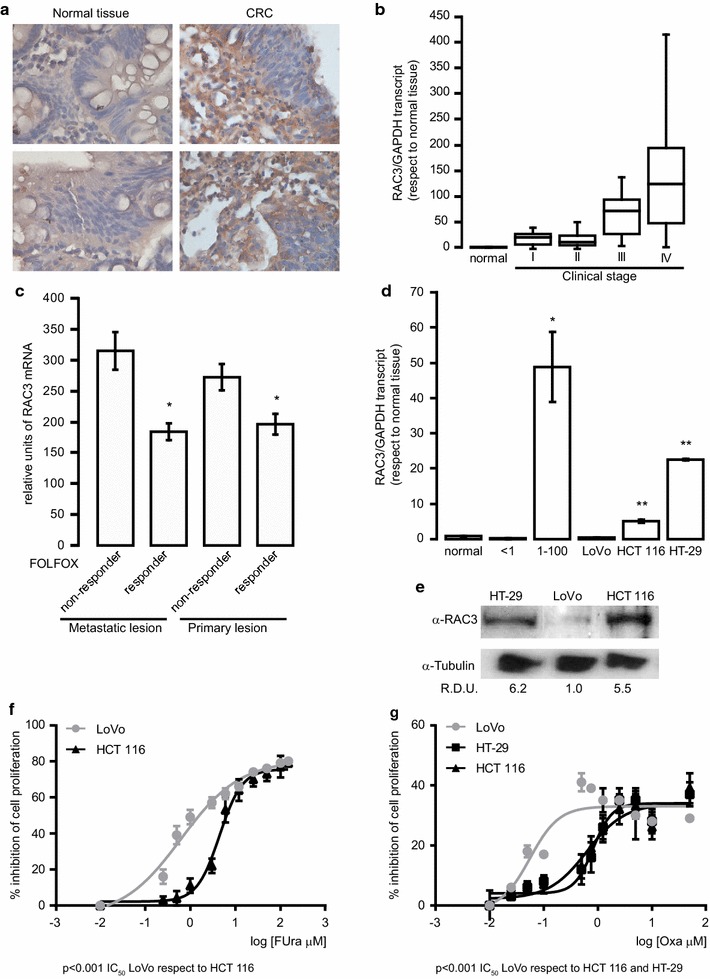
RAC3 acts as a predictive marker for CRC prognosis and chemotherapeutic response: **a** representative RAC3 Immunohistochemistry in normal colorectal and CRC tissues. **b** The RAC3 expression levels in normal colorectal tissues and CRC tissues at different pathological stages were determined by qPCR and normalized with GAPDH mRNA. **c** The RAC3 expression levels are compared between metastatic and primary lesion in patients sensitive or not to FOLFOX treatment (Folic acid-5-fluorouracil and oxaliplatin). The diagram bars show the average ± S.D. of mRNA expression log-transformed values from GSE28702 data bank, platform GPL570 Affymetrix (Santa Clara, CA, USA) *p < 0.01 respect to FOLFOX non-responder in Metastatic lesion. **d** RAC3 expression levels in three CRC cell lines are compared with normal colorectal and CRC tissues. The diagram bars correspond to average ± S.D. of RAC3 mRNA expression obtained by qPCR and normalized to GAPDH mRNA, *p < 0.001 respect to normal tissue and **p < 0.0001 respect to normal tissue and LoVo cell line. **e** Western blot was performed to determine the protein levels of RAC3 in the three CRC cell lines. Relative densitometry units (RDU) correspond to the average of densitometry units respect to Tubulin expression, obtained in three independent experiments. **f**, **g** Cell viability was determined by crystal violet staining and IC_50_ doses were calculated with GraphPad software. CRC cells were treated with FUra (0–150 μM) for 72 h (**f**) or Oxa (0–50 μM) for 24 h (**g**) p < 0.001 IC_50_ LoVo respect to HCT 116 to FUra or respect to HCT116 and HT-29 to Oxa

Firstly, we investigated the RAC3 expression levels in patients with colorectal cancer (CRC). Afterwards, the RAC3 mRNA expression was evaluated by qPCR assays in 17 exploratory biopsies, 14 of which were confirmed by pathological examination as CRC and 3 were normal tissues. RAC3 was found overexpressed in 11 (78%) of the 14 CRC biopsies and by immunohistochemical staining it was determined that the expression was mainly cytoplasmic (Table 1 and Fig. 1a). RAC3 expression was substantially higher in CRC than in normal colon tissue samples (p < 0.0294), whereas in studies that included a greater number of patients (n = 85), overexpression was observed in 35% of the samples [25]. Although a correlation between the clinical stage and the overexpression of RAC3 could be occurring, this hypothesis requires to be validated in larger cohorts of patients (Table 1 and Fig. 1b).

Then, we investigated RAC3 expression levels in metastatic and primary lesions from patients sensitive or not to FOLFOX (Folic acid-5-fluorouracil and oxaliplatin) treatment. We analyzed the DataSets records in the Gene Expression Omnibus (GEO) repository from GSE28702 data bank, platform GPL570 Affymetrix [31]. We found that patients who do not respond to FOLFOX treatment have higher coactivator expression levels than those patients that respond to treatment, being this difference significantly greater in metastatic lesions (Fig. 1c).

These results suggest that RAC3 could be probably considered as a predictive marker for CRC chemosensitivity.

### RAC3 expression levels in different CRC cell lines determine its sensitivity to chemotherapeutic drugs

5-Fluorouracil (FUra) and oxaliplatin (Oxa) are antimetabolite drugs that are widely used for cancer treatment, particularly for CRC [32]. Despite the increased understanding of the mechanism of action of these drugs, resistance to both of them remains as a significant limitation for its clinical use.

To explore the potential role of RAC3 in CRC sensitivity to drugs, we first investigated RAC3 expression in CRC cell lines (HT-29, HCT 116 and LoVo) by qPCR and western blot analysis. RAC3 expression levels were higher in HT-29 and HCT 116 cells than LoVo cells (Fig. 1d, e). Moreover, the RAC3 levels of this last cell line were similar to those expressed by normal colon tissues, as determined by qPCR (Fig. 1d), being not possible to confirm these results by western blot, due to the limiting amount of human colon normal tissues.

In view that these CRC cell lines express different levels of RAC3, they were employed as a model to validate the results obtained by GEO analysis. Thus, we investigated the sensitivity to FUra (0–150 μM) and Oxa (0–50 μM). Cell viability was determined by crystal violet staining and the IC_50_ was calculated for each cell type (Fig. 1f, g). We found that the cell line HT-29 overexpressing RAC3 did not respond to treatment with FUra in the concentration and time that we used. However, the LoVo cell line, whose RAC3 expression levels are lower, was more sensitive to treatment with these drugs (Table 2).

**Table 2:** Cytotoxic effects of 5-fluorouracil and oxaliplatin against colorectal cancer cell lines

	FUra		Oxa	
	IC_50_ μM (CI_95_)	% top inhibition (CI_95_)	IC_50_ μM (CI_95_)	% top inhibition (CI_95_)
HT-29	N.A.	N.A.	0.8 (0.6–1.0)	34% (31–36)
LoVo	0.6 (0.4–0.9)	80% (75–86)	0.05 (0.02–0.1)	33% (29–36)
LoVo control	0.6 (0.4–0.7)	82% (79–84)	0.04 (0.01–0.1)	30% (26–34)
LoVo RAC3	2.0 (1.4–2.8)	48% (45–51)	0.4 (0.25–0.6)	50% (46–53)
HCT 116 wt	4.5 (3.9–5.1)	75% (71–78)	0.6 (0.4–0.9)	36% (30–38)
HCT 116 control	4.2 (3.3–5.3)	78% (74–81)	0.6 (0.45–0.85)	42% (38–45)
HCT 116 shRAC3	2.4 (2.1–3.0)	88% (84–91)	0.17 (0.05–0.5)	52% (43–61)

Values represent results from at least three independent experiments

**Fig. 2:**
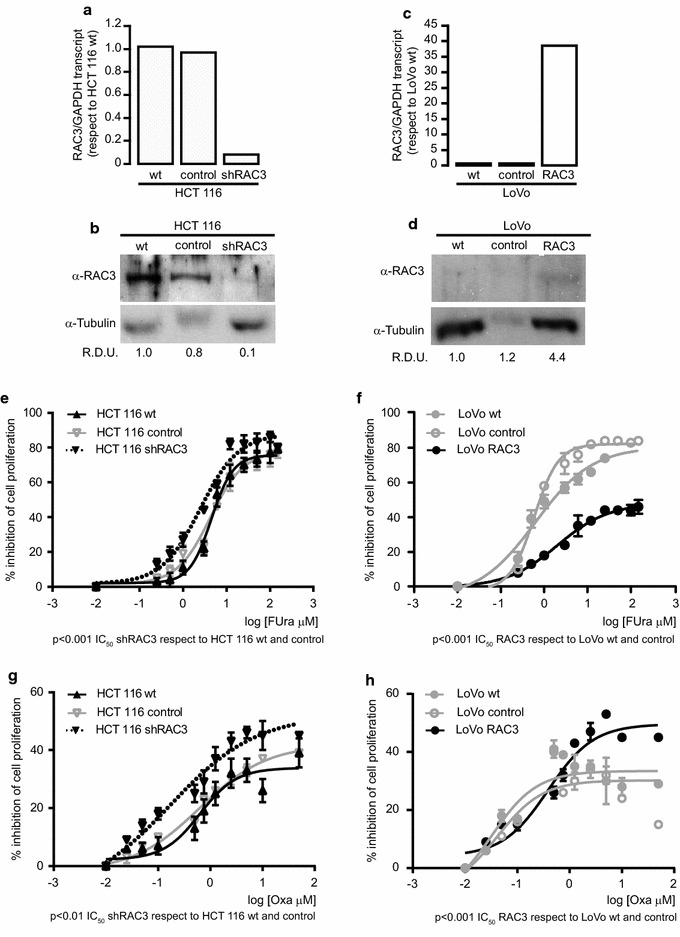
The levels of RAC3 expression affect the response to chemotherapeutic drugs: **a**–**d** Knocking down efficiency of shRAC3 in HCT 116 cell line (**a**, **b**) and overexpression of RAC3 in LoVo cells (**c**, **d**) were determined by qPCR normalized with GAPDH mRNA (**a**, **c**) and Western blot where RDU correspond to the average of densitometry units respect to the Tubulin expression, obtained in three independent experiments (**b**, **d**). **e**–**h** Cell viability was determined by crystal violet staining and IC_50_ doses were calculated with GraphPad software. CRC cells were treated with FUra (0–150 μM) for 72 h (**e**, **g**) or Oxa (0–50 μM) for 24 h (**f**, **h**) p < 0.001 IC_50_ HCT 116 shRAC3 or LoVo RAC3 respect to HCT 116 or LoVo wt and control for FUra in HCT 116 and LoVo and Oxa in LoVo cells and p < 0.01 IC_50_ HCT 116 shRAC3 respect to HCT 116 for Oxa

In order to determine whether the sensitivity observed in the different CRC lines could be dependent of the RAC3 expression levels, the HCT116 cell line was transfected with a plasmid containing the shRNA sequence for RAC3 (shRAC3) that we have previously used in other published works, having a good efficiency and specificity [28] or scramble sequence (control). Concerning the LoVo cells, that naturally express low RAC3 levels, they were transfected with aRAC3 expression vector or the empty vector (control) [28]. The efficiency of knockdown or overexpression was validated by western blot and qPCR (Fig. 2a–d).

We found that HCT 116 shRAC3 displayed a significantly decreased viability in response to chemotherapeutic drugs comparing to the control (Fig. 2e, g, Table 2). However, LoVo cells became more resistant to death-induced by both chemotherapeutic drugs when RAC3 is overexpressed (Fig. 2f, h, Table 2). Interestingly, the Oxa stimulation of this cell line follows a particular concentration-dependent response whose maximal proliferation inhibitory effect saturates reaching values that are below 40% in LoVo wt. However, although RAC3 overexpression significantly decreased the IC_50_, the curve of biological response was modified, reaching higher maximal values of cell death. This unexpected result for Oxa concentrations higher than 0.4 μM could be probably explained regarding the anti-senescent and proliferative stimulating role of RAC3 [33], which perhaps could be increasing the number of cells capable to respond to Oxa stimulation. Therefore, taking together all these results clearly demonstrate that the expression levels of RAC3 may influence the sensitivity to chemotherapeutic drugs, increasing the chemoresistance when is overexpressed.

### The decrease of RAC3 expression levels promotes apoptosis induced by FUra and Oxa

We have previously reported that overexpression of RAC3 inhibits apoptosis and this is one of the mechanisms involved in resistance to treatment with chemotherapeutic agents [26, 27]. Therefore, we decided to study whether RAC3 would be affecting the sensitivity to these chemotherapeutics via the inhibition of apoptosis. HCT 116, LoVo and HT-29 cell lines were stimulated with a drug concentration close to the IC_50_ and regarding the upper and lower limit of the CI_95_ for each cell line (Table 2).

**Fig. 3:**
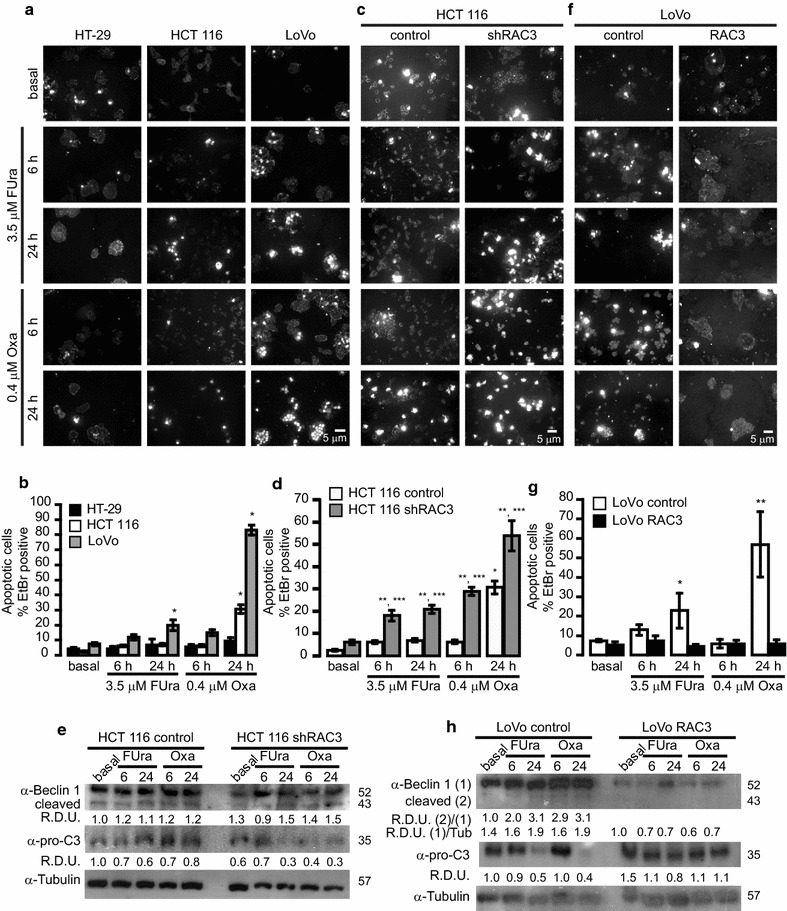
The sensitivity to drugs-induced cell death is enhanced by the decrease in RAC3 expression levels: Cells lines were stimulated for 6 or 24 h with 3.5 μM FUra or 0.4 μM Oxa. **a**, **c** and **f** Representative microphotography (200×) of apoptotic Ethidium Bromide (EtBr) stained cells after 6 or 24 h of treatment. **b**, **d** and **g** Diagram bars correspond to the percentage of EtBr positive cells per field (at least 10 fields per sample), **b** *p < 0.001 respect to basal condition for HCT 116 or LoVo cells, **d** *p < 0.001 respect to HCT 116 control basal, **p < 0.001 respect to HCT 116 shRAC3 basal and ***p < 0.001 respect to control and **g** *p < 0.05 respect to LoVo control basal, **p < 0.001 respect to LoVo control basal. **e** and **h** pro-Caspase 3 (pro-C3) and Beclin 1 levels were determined by Western blot. RDU correspond to the average of densitometry units respect to the Tubulin expression, obtained in three independent experiments

As shown in Fig. 3a, b and in agreement with that observed in assays of cell proliferation, HT-29 cells were not-sensitive to apoptosis induced by both drugs. However, LoVo cells were sensitive to Oxa and FUra-induced apoptosis after 24 h of treatment, showing a significant increase in the percentage of cells positive for Ethidium Bromide (EtBr) (Fig. 3a, b). In the case of HCT 116, only Oxa was able to induce apoptosis after 24 h.

Therefore, using the cells where apoptosis could be induced, we then investigated if this process could be affected by different RAC3 expression levels. Thus, we found that sensitivity to apoptosis of HCT 116 cells was significantly enhanced when RAC3 was knocked (shRAC3), showing a significant increase of positive apoptotic cells after 6 h of treatment, earlier than HCT 116 control cells (Fig. 3c, d). Moreover, they became sensitive to FUra-induced apoptosis.

In agreement with previous evidences, the active Caspase 3 is an indicator of late apoptosis, while Beclin 1 is one of its targets. In addition, the cleavage products of this last protein inhibit autophagy but promote apoptosis [34]. Then, we analyzed the modulation of these proteins by drugs stimulation in cells overexpressing or not RAC3. We found a significant reduction of pro-Caspase 3, accompanied by the increased Beclin 1 cleavage in cells with low RAC3 expression, after treatment with FUra for 24 h (RDU 1.5 vs 1.1) and Oxa at 6 (1.4 vs 1.2) or 24 h (1.5 vs 1.2), (Fig. 3e).

In order to confirm these results we then investigated if LoVo cells, which naturally express low levels of RAC3 and are sensitive to apoptosis induced by both drugs, may be affected by RAC3 overexpression. Therefore, we analyzed apoptosis in control and RAC3 overexpressing cells performing the same experiments as in HCT 116 cells. As shown in Fig. 3f, g, when LoVo cells overexpress RAC3 they became completely resistant to both drugs, at least in the concentrations that were assayed. As expected, no cleavage of pro-Caspase 3 and Beclin 1 could be detected (Fig. 3h).

Therefore, our results suggest that expression levels of RAC3 could be affecting the sensitivity to apoptosis-induced by chemotherapeutic drugs; however, autophagy could be also involved.

### The increase of autophagy under low RAC3 expression levels maximizes the FUra treatment

Recent studies suggest that autophagy could be playing an important role in cancer development, determining the response to anticancer therapy. However, the role of autophagy in these processes is not at all clear, thus, depending on the circumstances, it may have diametrically opposite consequences for the tumor [35].

Many anticancer agents have been reported to induce autophagy, supporting the idea that autophagic cell death may be an important mechanism for tumor cell killing by these agents [36].

We have previously demonstrated that RAC3 overexpression inhibits autophagy [28]. Therefore, in order to investigate whether RAC3 could be affecting autophagy CRC cell lines, we first investigated if these drugs are capable to induce this response.

**Fig. 4:**
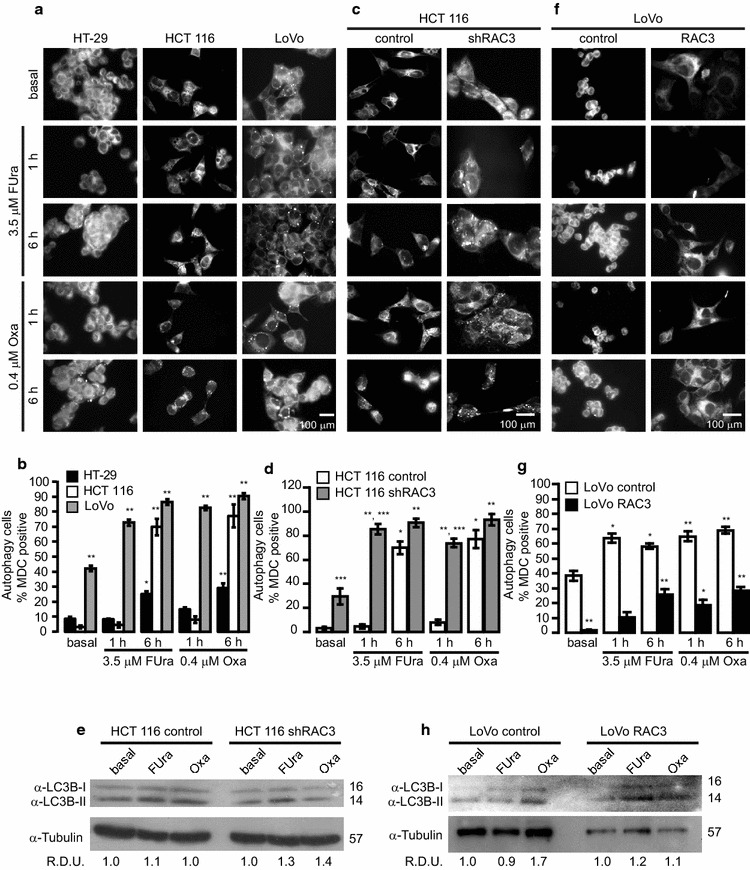
Autophagy induction by FUra and Oxa is dependent of RAC3 levels: Cells lines were loaded in 24 well plates with slices and after 24 h cells were stimulated with 3.5 μM FUra or 0.4 μM Oxa. **a**, **c** and **f** Autophagy was determined by staining with monodansylcadaverine (MDC) after 1 or 6 h post-treatment. **b**, **d**, **g** Diagram bars correspond to percentage of MDC positive cells per field (at least 10 fields per sample). Statistical analysis ANOVA and Tukey post-test n = 3 were performed, **b** *p < 0.01 3.5 μM FUra at 6 h HT-29 respect to HT-29 basal, **p < 0.001 LoVo basal respect to HT-29 and HCT 116 basal, LoVo treated at each time respect to LoVo basal, HT-29 0.4 μM Oxa at 6 h respect to HT-29 basal and HCT 116 treated for 6 h with FUra or Oxa respect to HCT 116 basal. **d** *p < 0.001 3.5 μM FUra and 0.4 μM Oxa at 6 h HCT 116 control respect to HCT 116 control basal, **p < 0.001 HCT 116 shRAC3 treated respect to shRAC3 basal, ***p < 0.001 shRAC3 respect to control and **g** *p < 0.01 3.5 μM FUra LoVo control respect to Lovo control basal and LoVo RAC3 treated with 0.4 μM Oxa for 1 h respect to LoVo RAC3 basal, **p < 0.001 LoVo RAC3 basal respect LoVo control basal, 3.5 μM FUra at any time LoVo control respect LoVo control basal and 0.4 μM Oxa LoVo RAC3 at 6 h respect to LoVo RAC3 basal. **e**, **h** LC3-II/I levels were determined by Western blot. RDU correspond to the average of densitometry units, respect to Tubulin expression

Thus, cell lines were stimulated with FUra (3.5 μM) or Oxa (0.4 μM) for 1 or 6 h. The Fig. 4a, b show that both drugs may induce autophagy in all the cell lines, including HT-29 cells which are resistant to apoptosis. In LoVo cells, the basal autophagy was naturally high, suggesting it could be a surviving strategy as previously described [28]. In the case of HT29 cells, although both drugs were unable to induce cell death (Fig. 3b), they trigged autophagy, suggesting it plays a surviving role, as in LoVo cells [28].

Being these cells sensitive to autophagy induced by both drugs, we then analyzed how could be affected by RAC3.

We found that autophagy started earlier (1 vs 6 h) and was significantly higher in HCT 116 shRAC3 than in control HCT 116 (Fig. 4c–e). In the case of LoVo cells, the RAC3 overexpression significantly inhibited the autophagy induced by both drugs, as determined by MDC staining and LC3 detection (Fig. 4f–h).

Hence, in agreement with these results, we found that FUra was a better inducer of autophagy than apoptosis in cells that overexpress RAC3, suggesting this is the main mechanism by which induces cell death. However, the anti-tumoral effect of Oxa shows to be mainly mediated through apoptosis.

The sensitivity to both drugs was significantly increased under low expression levels of RAC3, showing a greater apoptosis and autophagy which were evidenced earlier than in cells overexpressing RAC3.

### The oxaliplatin and 5-fluorouracil—induced decrease of acetylated proteins is enhanced in cells expressing low levels of RAC3

Accumulating evidence suggests that the long-term success of anti-neoplastic therapies is largely determined by their capacity to reinstate anticancer immunosurveillance [37]. One of the requisites of immunogenic chemotherapy is the induction of autophagy [38]. This process allows the optimal lysosomal exocytosis of ATP from dying tumor cells [39] and avoids the up regulation of the immunosuppressive ecto-ATPase CD39 [40].

**Fig. 5:**
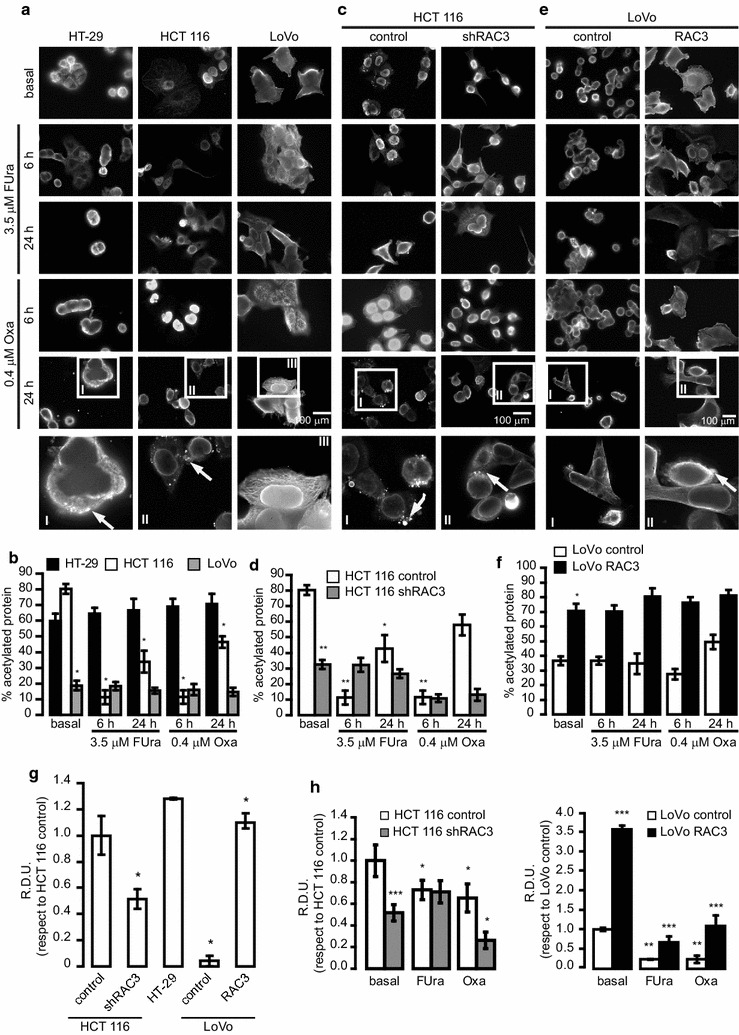
Autophagy induction by FUra and OXA induces a decrease in protein acetylation in HCT 116 cells: **a**, **c** and **e** Cells were pre-treated with a deacetylase inhibitor TSA (0.4 μM) for 30 min and then, stimulated with FUra (3.5 μM) or Oxa (0.4 μM) for 6 or 24 h. The arrows show the positive acetylation detected by immunofluorescence using an anti-Lysine acetylated antibody and an antibody anti-mouse coupled to Rodamine. A cell detail of the 10 × magnification is shown in the square. **b**, **d** and **f** Diagram bars correspond to percentage of acetylated protein per field (at least 10 fields per sample). Statistical analysis ANOVA and Tukey post-test n = 3 were performed. **b** *p < 0.001 LoVo TSA respect to HT-29 and HCT 116 TSA, HCT 116 treated respect to HCT 116 TSA, **d** *p < 0.01 HCT 116 control TSA plus 3.5 μM FUra at 24 h respect to HCT 116 control TSA, **p < 0.001 HCT 116 control TSA plus FUra or Oxa at 6 h respect HCT 116 control TSA and HCT 116 shRAC3 TSA respect HCT 116 control TSA and **f** *p < 0.001 LoVo RAC3 with TSA respect to LoVo control TSA. **g** Expression levels of CD39 in CRC cell lines. The diagram bars show the average ± S.D. of CD39 expression normalized to GAPDH from three independent experiments, *p < 0.001 HCT 116 shRAC3 and LoVo control respect to HCT 116 control and Lovo RAC3 respect to LoVo control. **h** The diagram bars show the average ± S.D. of CD39 expression normalized to GAPDH from three independent experiments, *p < 0.001 HCT 116 shRAC3 basal respect to HCT 116 control basal and **p < 0.01 cells treated with 0.4 μM Oxa respect to HCT 116 control basal or HCT 116 shRAC3 basal

In view that reduction in lysine acetylation of cellular proteins could be a good inducer of autophagy and ATP exocytosis [40], we investigated the effect of FUra and Oxa over the degree of protein acetylation. Although HCT 116 and HT-29 cell lines have similar basal levels of acetylated proteins, we found that acetylation was significantly inhibited by drugs stimulation only in HCT116 (Fig. 5a, b), Interestingly, this cell line is which better respond to autophagy induction (Fig. 4a, b).

Concerning LoVo cells, which naturally express low levels of RAC3, the basal acetylation was lower than in the other cell lines and no modulation by drugs stimulation was observed (Fig. 5a, b).

When we analyzed the effect of RAC3 knocking in HCT 116, we found that both basal and drug-induced acetylation at 24 h were significantly inhibited respect to HCT116 control cells (Fig. 5c, d).

In the case of LoVo cells, RAC3 overexpression induced a significant increase of basal acetylation that could not be inhibited by anyone of the drugs (Fig. 5e, f).

Taken together all these results, we may conclude that high RAC3 expression contributes to the increased basal levels of protein acetylation, which could be compatible with its intrinsic histone acetylase activity [16], although additional substrates could not be excluded. However, different levels of RAC3 expression do not modify the sensitivity to drugs-induced acetylation. Therefore, despite acetylation inhibition could be involved in autophagy induction, our results demonstrate that this is not the mechanism by which RAC3 may modulate the sensitivity to drugs-induced autophagy.

Despite pre-mortem autophagy is not essential for chemotherapy-elicited cancer cell death to occur [41], it is indispensable for immunogenic cell death (ICD) through the ATP release into the extracellular space where it serves as a chemotactic factor to attract antigen-presenting cells into the neighbor microenvironment of dying cells [38, 42]. This effect is achieved by the capacity of extracellular ATP to act on purinergic receptors in the surface of immature dendritic cell precursors and T cells [42]. The suppression of autophagy in tumor cells or CD39 overexpression by tumoral cells, blockade the capacity of chemotherapy to stimulate the invasion of tumors by antigen-presenting cells [42].

Since cells overexpressing RAC3 have a low drug-induced autophagy, we analyzed the CD39 expression in cells having high and low RAC3 levels. We found that without stimulation, those cells having low RAC3 (LoVo control and HCT 116 shRAC3) show a lower expression of CD39 than cells with high coactivator expression (HT-29, LoVo RAC3 and HCT 116 control) (Fig. 5g). Then, we investigated the effect of Oxa and FUra over HCT 116 and LoVo. We found that Oxa induced a significant decrease of CD39 in control and shRAC3 HCT116 although not FUra effect was observed in shRAC3 (Fig. 5h). In the case of LoVo cells, both drugs induced the CD39 inhibition (Fig. 5h).

Therefore, although RAC3 did not affect the natural biological action of these drugs, cells expressing low RAC3 were more sensitive to chemotherapeutics-induced autophagy and express lower levels of CD39.

### Chemoresistance under high RAC3 expression levels could be partially mediated through the enhanced NF-κB activity

We have previously demonstrated that RAC3 is a NF-κB coactivator [23] that exerts an anti-apoptotic and anti-autophagic role when is overexpressed, not only enhancing the expression of NF-κB target genes, but also through additional cytoplasmic actions [27, 28]. Moreover, the overexpression of a mutated form of RAC3 lacking the tag nuclear signal, which avoids its nuclear translocation, also exerts an anti-apoptotic role [28].

Therefore, in view that RAC3 is mainly localized in the cytoplasmic compartment of CRC, we investigated if at least part of the chemoresistance to FUra and Oxa-induced cell death could be mediated through NF-κB activity.

**Fig. 6:**
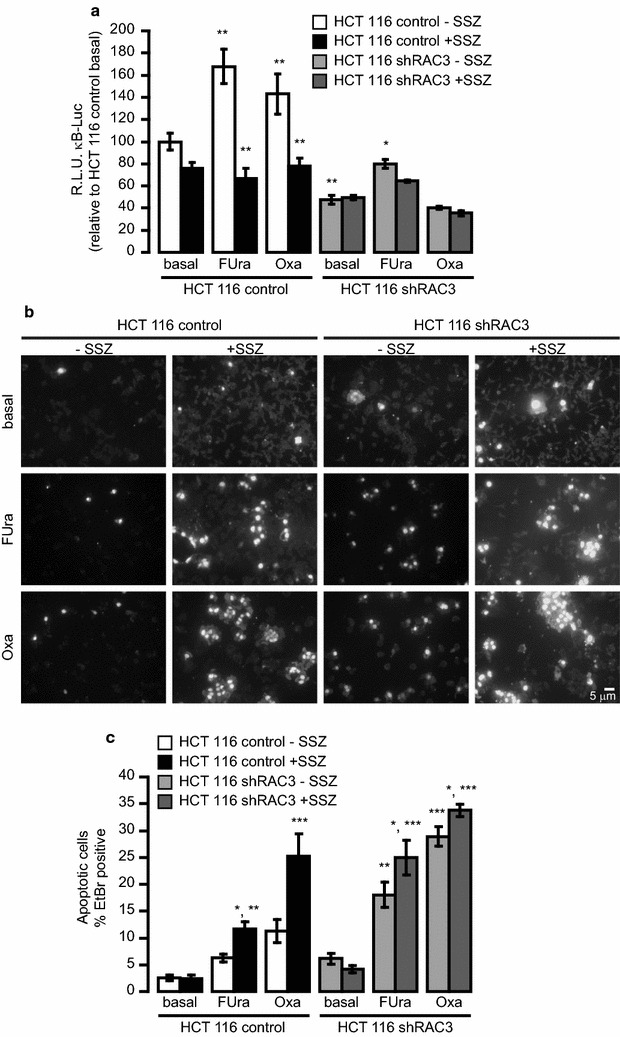
NF-κB activity is partially involved in the chemoresistance induced by high RAC3 expression levels: **a** HCT 116 control or shRAC3 cells were co-transfected with κB-Luc reporter plasmid plus RSV-β Gal. Cells were pre-stimulated for 30 min with 0.5 mM SSZ or DMSO, prior to treatment with 3.5 μM FUra or 0.4 μM Oxa for 24 h. The diagram bars correspond to the average ± S.D. of relative light units normalized with the corresponding β-galactosidase values, *p < 0.01 HCT 116 shRAC3 treated with 3.5 μM FUra respect to HCT 116 shRAC3 basal and ***p < 0.001 HCT 116 control treated with drugs respect to HCT 116 control basal, HCT 116 control treated with FUra or Oxa plus SSZ respect to HCT 116 control without SSZ and HCT 116 shRAC3 respect to HCT 116 control. **b**, **c** HCT 116 cell lines were pre-stimulated for 30 min with 0.5 mM SSZ and then the cells were treated for 6 h with 3.5 μM FUra or 0.4 μM Oxa. **b** Representative microphotography (200×) of apoptotic Ethidium Bromide (EtBr) stained cells after 6 h of treatment and **c** Diagram bars correspond to the percentage of EtBr positive cells per field (at least 10 fields per sample), *p < 0.05 HCT 116 control or shRAC3 FUra plus SSZ respect to HCT 116 treated with FUra and HCT 116 shRAC3 Oxa plus SSZ respect to shRAC3 Oxa, **p < 0.01 HCT 116 control FUra + SSZ respect to HCT 116 control basal and HCT 116 shRAC3 FUra respect to HCT 116 hsRAC3 basal, ***p < 0.001 HCT 116 control Oxa + SSZ respect to HCT 116 control basal and Oxa, HCT 116 shRAC3 FUra + SSZ or Oxa + SSZ respect to HCT 116 shRAC3 treated with FUra or Oxa, respectively

First, we investigated if FUra and Oxa could induce the NF-κB activation at the concentrations that were assayed in all the experiments. We found that both drugs were capable to activate NF-κB in control cells, as expected for several stress signals or apoptosis inducers [30]. This activity was significantly inhibited by the IKK inhibitor SSZ showing values similar to basal conditions (Fig. 6a). However, the drugs-induced NF-κB activity was significantly lower in shRAC3 than in control cells, as expected, being RAC3 a required NF-κB coactivator [23]. Therefore, no effect of SSZ could be detected over drugs-induced NF-κB activity in shRAC3 cells (Fig. 6a).

Then, using the same SSZ concentration, we investigated its effect over drugs-induced apoptosis. As shown in Fig. 6b, c, SSZ significantly increased the FUra and Oxa-induced apoptosis in both control and shRAC3 HCT 116. Although the increased cell death in control HCT 116 was almost 100%, a lower effect but still significant was observed in HCT 116 shRAC3. Therefore, most of the resistance of RAC3-overexpressing cells to chemotherapeutics-induced apoptosis could be mainly mediated through an increased NF-κB activity. However, our results also suggest that additional signals not related to this transcription factor but at least dependent of IKK activity could be involved.

## Discussion

Previous studies demonstrated that RAC3 is a critical predictor of cancer prognosis in different cancer types [43, 44]. The gene for this molecule was reported to be amplified in 2–10% of human breast tumors and the protein overexpressed in 30–60% of tumors, suggesting that RAC3 provides a growth advantage for breast cancer cells [44]. In CRC studies, a copy number gain of RAC3 was detected in 27.5% and it is overexpressed in 35% CRC samples correlating with tumor progression [25, 45]. Despite all these evidences, the role of RAC3 in CRC chemoresistance is still unknown.

In the present study, we reported new findings compelling evidence that RAC3 overexpression in CRC cell lines decreases the sensitivity to chemotherapeutic drugs and the mechanism involves the inhibition of apoptosis and autophagy induced by these agents.

In this work, we found that normal tissues express low levels of RAC3, but CRC samples shown an overexpression of this molecule, as expected, while its localization was mainly cytoplasmic. This last observation could be particularly interesting concerning the mechanisms by which RAC3 may contribute to CRC development. Indeed, it is in agreement with our previous works demonstrating that besides its nuclear role as a coactivator, enhancing the NF-κB activity and increasing the anti-apoptotic genes expression, RAC3 has a cytoplasmic role, regulating kinase activity, interacting with cytoskeletal proteins, delaying the AIF nuclear translocation and inhibiting apoptosis [27].

Concerning the RAC3 anti-autophagic role, we have previously demonstrated that part of the mechanism involves NF-κB activation. However, the overexpression of a truncated form of RAC3 that lacks the signal for nuclear translocation also exerts an anti-autophagic role, suggesting that cytoplasmic actions of RAC3 may contribute to this activity [28]. Therefore, RAC3 is a very particular molecule which has the ability to modulate multiple biological responses through several not related mechanisms in different cellular compartments. Thus, in the nucleus it can modify the transcriptional activity of steroid nuclear receptors and transcription factors through its own histone acetylase activity, but also by recruitment of other molecules, changing the expression pattern of a wide amount of target genes [16]. In addition, in the cytoplasm, RAC3 may be associated to several proteins modulating its activity [27]. Although no cytoplasmic RAC3 acetylase substrates have been identified to date, this possibility should not be excluded and deserves to be investigated, regarding the RAC3 cytoplasmic abundance in some tumors, as well as our own results concerning the increase of total proteins acetylation when RAC3 is overexpressed.

Afterwards, the predominantly cytoplasmic localization observed in CRC tissues could be determinant in chemotherapeutic response and probably related to the molecular mechanisms of both apoptosis and autophagy signaling pathways where RAC3 exerts the inhibitory effect. However, our results demonstrate that despite its cytoplasmic localization, most of the anti-apoptotic effect under chemotherapeutics treatment could be mediated through an enhanced NF-κB activity, as a nuclear receptor coactivator. However, additional signals, nuclear or cytoplasmic, could not be excluded in the RAC3-induced anti-apoptotic and anti-autophagic activity.

In a previous work performed in primary cutaneous melanoma samples, the authors described RAC3 as a prognostic marker for the prediction of survival associated with melanoma [46], while in breast cancer the RAC3 overexpression was considered as a predictor of resistance to Tamoxifen treatment [44]. In this regard, in this work, through the in silico analysis we demonstrate a correlation between the response to FOLFOX treatment and the RAC3 expression levels. Therefore, our findings in CRC are in agreement with other previous works, supporting that levels of RAC3 expression could have a possible predictive role concerning chemoresistance.

When we analyzed the expression levels of RAC3 in patients of the IDIM hospital population and its correlation with the tumor stage, the results obtained show a tendency similar to that reported by other authors [25]. Because our work involves exclusively an aged population, it is particularly interesting, given that a higher CRC incidence in patients over 65 has been described in last recent years [47]. However, preventive care in this population has only been addressed by a limited number of guidelines and cancer screening in the elderly (those greater than 75 years of age) has been controversial [47, 48].

The lifetime risk of colorectal cancer is ~ 5% with an incidence in the population over 75 years at about 40–50 per 100,000 persons. However, the incidence falls off to 15–20 per 100,000 in persons 60–65 years [2]. Therefore, although we have not performed a comparative study between young and elderly patients, our findings contribute to increase the knowledge about molecules to be studied in CRC, not only as possible predictive markers of chemoresistance, but also as targets in order to improve anticancer therapies.

As discussed above, although the inhibitory effects of RAC3 overexpression on the action of chemotherapeutic drugs have been investigated, most of these studies are restricted to its role as a coactivator in the nucleus. We have previously demonstrated that RAC3-overexpressing cells are resistant to apoptosis and furthermore, RAC3-knocked K562 leukemic cell line becomes sensitive to treatment with flavopiridol [26, 27]. Indeed, in this work, we demonstrated for the first time that while RAC3 overexpression inhibits 5-fluorouracil and oxaliplatin-induced apoptosis, its knocking turns CRC cells into sensitive.

Several works support a dual role for autophagy in tumor development: Inducing cell death, or exerting a surviving role [49]. This last role is in agreement with the elevated basal autophagy levels usually found in tumoral cells, like pancreatic cancer [50] as well as the surviving under stress conditions like hypoxia and starving [51, 52], a common situation to which cancer cells are exposed before the angiogenesis, migration and invasion. However, it is also known that an exacerbated autophagy could lead to cell death, and moreover, this could be the mechanism by which some chemotherapeutic drugs attack the tumoral cells [36].

Despite the controversial role of autophagy in cancer development and tumor progression, recent studies demonstrate that autophagy has a relevant function in chemotherapeutic efficiency by induction of ATP exocytosis, chemotaxis of dendritic cells and ICD induction [39]. In this work, we demonstrate that both 5-fluorouracil and oxaliplatin are able to induce autophagy more efficiently in cells that express low levels of RAC3 through at least, a diminished protein acetylation and CD39 down regulation. Interestingly, both processes are involved in the capacity to stimulate the invasion of tumors by antigen-presenting cells.

## Conclusions

Taken all together these results, we demonstrate for the first time that RAC3 overexpression could be playing a critical role in CRC tumoral cells affecting the sensitivity to apoptosis and autophagy induced by chemotherapeutic drugs. Moreover, concerning the mechanism, our results demonstrate that at least part of the chemoresistance due to RAC3 overexpression could be dependent of its transcriptional activity as coactivator of NF-κB. However, in agreement with our results showing a strong RAC3 cytoplasmic localization in CRC cells, additional not-nuclear effects could not be excluded, being perhaps an original and interesting point to be considered in order to design future improved therapies for CRC treatment.

## Abbreviations

AIB-1: amplified in breast cancer-1
AIF-1: apoptosis-inducing factor-1
CRC: colorectal cancer
GEO: Gene Expression Onmibus
FOLFOX: folic acid-5-fluorouracil and oxaliplatin
FUra: 5-fluorouracil
IDC: immunogenic cell death
MDC: monodansylcadaverine
Oxa: oxaliplatin
RAC3: receptor associated-coactivator 3
TSA: trichostatin A
